# An Expeditious Neutralization Assay for Porcine Reproductive and Respiratory Syndrome Virus Based on a Recombinant Virus Expressing Green Fluorescent Protein

**DOI:** 10.3390/cimb46020066

**Published:** 2024-01-23

**Authors:** Juan Wang, Jiecong Yan, Shuaiyong Wang, Ronglin Chen, Yanru Xing, Qingyan Liu, Shuolei Gao, Yuxiang Zhu, Jiannan Li, Yanjun Zhou, Tongling Shan, Wu Tong, Hao Zheng, Ning Kong, Yifeng Jiang, Changlong Liu, Guangzhi Tong, Hai Yu

**Affiliations:** 1 Shanghai Veterinary Research Institute, Chinese Academy of Agricultural Sciences, Shanghai 200241, China; juanwang202312@163.com (J.W.);; 2 Jiangsu Co-Innovation Center for Prevention and Control of Important Animal Infectious Diseases and Zoonoses, Yangzhou 225009, China

**Keywords:** PRRSV, infectious clones, reporter virus, enhanced GFP, neutralizing antibodies, neutralization assay

## Abstract

Due to the extensive genetic and antigenic variation in Porcine Reproductive and Respiratory Syndrome Virus (PRRSV), as well as its rapid mutability and evolution, PRRS prevention and control can be challenging. An expeditious and sensitive neutralization assay for PRRSV is presented to monitor neutralizing antibodies (NAbs) in serum during vaccine research. Here, a PRRSV expressing eGFP was successfully rescued with reverse genetics based on the infectious clone HuN4-F112-eGFP which we constructed. The fluorescent protein expressions of the reporter viruses remained stable for at least five passages. Based on this reporter virus, the neutralization assay can be easily used to evaluate the level of NAbs by counting cells with green fluorescence. Compared with the classical CPE assay, the newly developed assay increases sensitivity by one- to four-fold at the early antibody response stage, thus saving 2 days of assay waiting time. By using this assay to unveil the dynamics of neutralizing antibodies against PRRSV, priming immunity through either a single virulent challenge or only vaccination could produce limited NAbs, but re-infection with PRRSV would induce a faster and stronger NAb response. Overall, the novel HuN4-F112-eGFP-based neutralization assay holds the potential to provide a highly efficient platform for evaluating the next generation of PRRS vaccines.

## 1. Introduction

Porcine Reproductive and Respiratory Syndrome (PRRS) is a highly contagious infection that affects domestic pigs of all ages. This disease, characterized by reproductive failure in sows and respiratory distress in growing pigs, poses a significant danger to the pork industry on a global scale [1,2]. There are two genotypes of PRRSV: the European type (Type 1) and the North American type (Type 2) [3]. The similarity between the genomes of these two virus types is approximately 65% [4,5]. In 2006, there was an outbreak of Highly Pathogenic Porcine Reproductive and Respiratory Syndrome (HP-PRRS) in China [6,7]; subsequently, in 2013, the PRRSV NADC30 strain was introduced to China [8]. The NADC30 strain has since recombined with HP-PRRSV, which has made the disease’s epidemiology very intricate [9].

It is well known that vaccines are a powerful tool for preventing and controlling infectious diseases [10]. Due to the extensive genetic and antigenic variation in PRRSV, as well as its rapid mutability and evolution, a variety of emerging and re-emerging virus strains have continued the emergence of worldwide epidemics [11,12]. Therefore, PRRS prevention and control can be challenging, although several PRRSV vaccines have been developed [13,14]. A study by Lopez et al. confirmed that PRRSV-inactivated vaccines that did not produce NAbs did not offer protection, while attenuated vaccines that generated some level of NAbs were able to provide protection [15]. Natural antibodies provide instant protection for animals at high risk of infection, which is key in preventing PRRSV infection [15,16]. Therefore, measuring the NAb titer could serve as a useful factor in evaluating the efficacy of PRRSV vaccines [17]. 

There are two main types of classical neutralization antibody tests: cytopathic effect (CPE) [18,19] and fluorescent focus neutralization (FFN) assays [20,21]. While these two methods are commonly utilized in diagnostic laboratories, they do have certain limitations: (1) the sensitive PRRSV-specific antibody used for IFA detection is difficult to obtain and expensive; (2) both of these assays are time-consuming, as it takes four days to obtain a diagnosis of cytopathy with a CPE assay, while the FFN assay utilizes the time-consuming process of an indirect immune fluorescent assay (IFA) [22]. By contrast, neutralization assays based on reporter viruses can save time, simplify the experimental process, and provide sensitive and reliable results [23,24]. With the development of virus reverse genetics technologies for PRRSV, PRRSV has been used as a viral vector for expressing a desired foreign gene. Reporter genes such as luciferase [23,25] and clover [24] can be used to track viral infection. Cytokines (IL-15, IL-18, GM-CSF, etc.) were inserted in PRRSV genomes to improve vaccine efficacy [26,27,28]. In addition, certain virus antigenic proteins, such as the E2 protein of Classical Swine Fever Virus (CSFV) [29] and the S protein of G2 Porcine Epidemic Diarrhea Virus (PEDV) [30], were incorporated into PRRSV genomes in order to create a bivalent vaccine.

In this study, the enhanced GFP gene (eGFP), fused with PRRSV TRS2, was inserted at the C terminus of the ORF1b gene in the HuN4-F112 infectious clone. Then, we successfully rescued a stable virus strain expressing the eGFP, based on the infectious clone HuN4-F112-eGFP. The novel HuN4-F112-eGFP-based neutralization assay holds the potential to provide a highly efficient platform for evaluating the next generation of PRRS vaccines.

## 2. Results

### 2.1. Generation of an Infectious Clone and Reporter Virus

**Figure 1:**
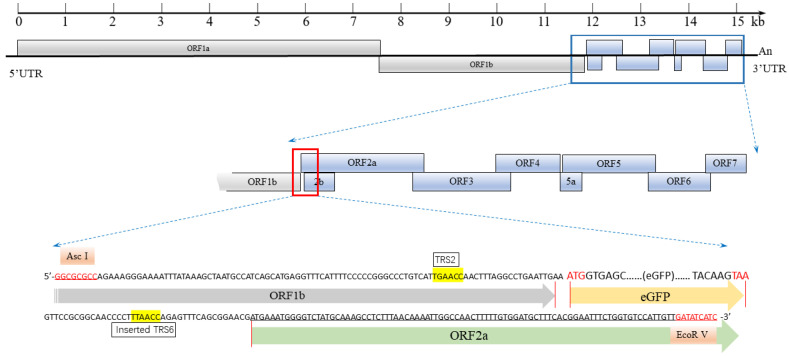
Schematic diagram of the HuN4-F112 infectious clone with the eGFP gene inserted between open reading frame 1b (ORF1b) and ORF2a. The eGFP gene was flanked by the Asc I and EcoR V sites for insertion. The red letters is DNA sequences of the two restriction sites (Asc I and EcoR V). After digestion with Asc I and EcoR V, the eGFP gene, fused with PRRSV TRS2, was inserted at the C terminus of the ORF1b gene in the HuN4-F112 infectious clone. The recombinant PRRSV full-length cDNA clone harboring eGFP was named HuN4-F112-eGFP.

**Figure 2:**
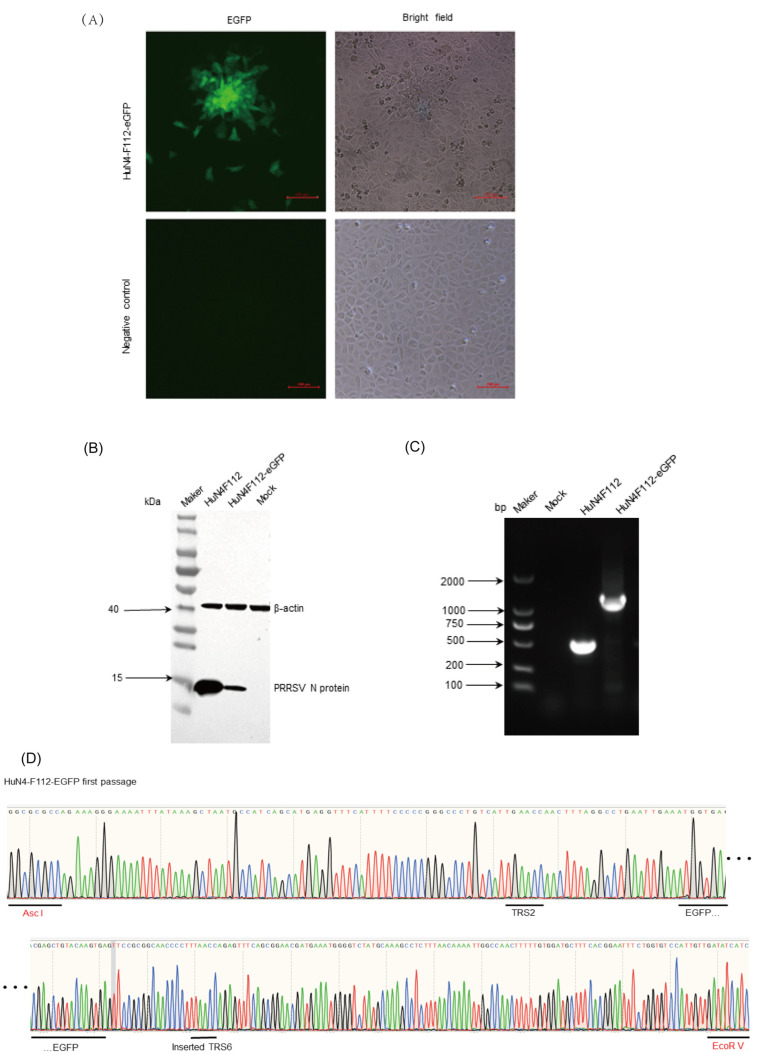
Recovery and identification of the recombinant virus HuN4-F112-eGFP. (**A**) The cytopathic effect (CPE), characterized by cellular rounding and clumping, was observed 4 days post-transfection. At the same position, the fluorescence expression was monitored under an inverted fluorescence microscope. Scale bar: 100 µm. (**B**) MARC-145 cells were analyzed using Western blotting to detect PRRSV N proteins at 24 h post-infection. (**C**) PCR confirmation of recombinant plasmids HuN4-F112-eGFP with F1 and R1. The HuN4-F112-eGFP and parental HuN4-F112 were, respectively, used as the cDNA to amplify the insert fragments. (**D**) Sequencing result of the eGFP gene in HuN4-F112-eGFP for the first passage. The underlinings marked the DNA sequences of the special fragments in the sequencing result. The Asc I and EcoR V were the restriction sites.

The synthesized fragment harboring eGFP was inserted between ORF1b and ORF2 of the full-length cDNA clone of HuN4-F112, according to the proposed strategy (Figure 1). The recombinant PRRSV with eGFP was named HuN4-F112-eGFP. HuN4-F112-eGFP was used for in vitro transcription by SP6 RNA polymerase. MARC-145 cells were transfected with the synthesized RNA with the transfection reagent DMRIE-C. The cytopathic effect (CPE), characterized by cellular rounding and clumping, was observed 4 days post-transfection (Figure 2A). At the same position, fluorescence expression was monitored under an inverted fluorescence microscope (Figure 2A). The control MARC-145 cells had no CPE or fluorescence (Figure 2A). The expressions of N proteins of the recombinant PRRSV were confirmed through Western blotting with PRRSV N-specific antibodies (Figure 2B). The PCR products of HuN4-F112-eGFP and parental HuN4-F112, using F1 and R1 primers, were strands of unequal length (Figure 2C). Furthermore, the eGFP tags were confirmed to exist when sequencing the PCR products, which were found to be consistent with the original sequences, with no additional mutations introduced (Figure 2D).

### 2.2. Biological Characteristics of HuN4-F112-eGFP

**Figure 3:**
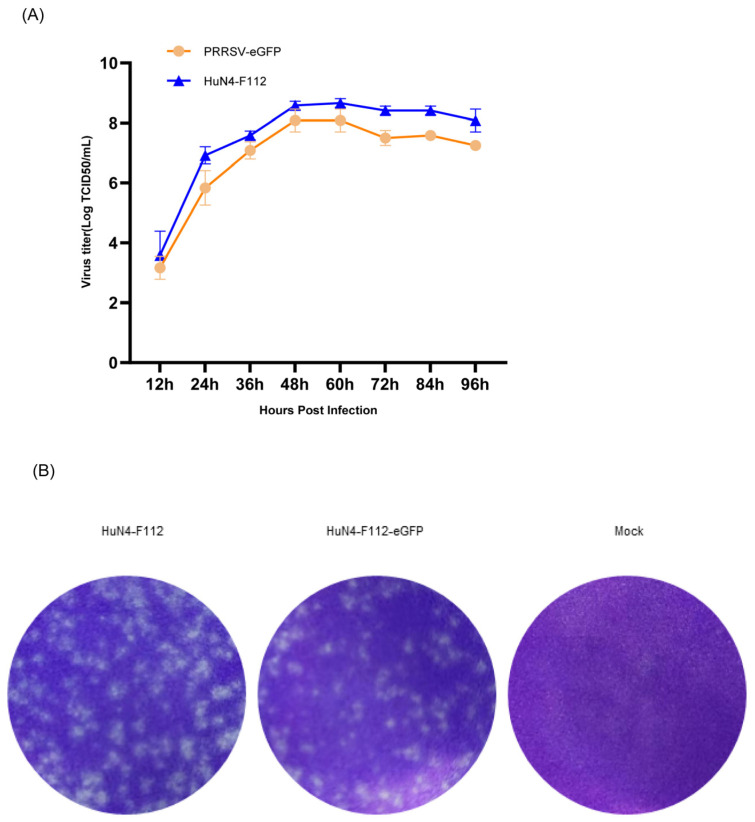
Biological characteristics of HuN4-F112-eGFP. (**A**) Comparison of virus multistep growth curves of HuN4-F112-eGFP and parental HuN4-F112. MARC-145 cells were infected with the recombinant viruses (P5) and the parental viruses at an MOI of 0.01. Virus supernatants, collected at 12 h intervals from 0 to 96 hpi, were measured using the TCID_50_ assay. Each data point represents the mean ± deviation of duplicates. (**B**) Viral plaque morphology assays comparing HuN4-F112-eGFP to parental HuN4-F112. MARC-145 cells were infected with serial diluted recombinant viruses and covered with 1% low-melting-point agarose supplemented with 2% FBS. At 3 days post-infection, the plaques formed by virus infection were visualized using crystal violet staining.

Virus multistep growth curves of HuN4-F112-eGFP and parental HuN4-F112 were determined for MARC-145 cells to determine the effect of eGFP gene insertion. The eGFP expression had some influence on the growth characteristics of the rescued virus. The peak titer of HuN4-F112-eGFP was slightly lower than those of the parental viruses, although the titers peaked at 48 hpi for both viruses (Figure 3A). As expected, the results of plaque morphology experiments showed that the plaques of HuN4-F112-eGFP were smaller than those of the parental viruses (Figure 3B).

### 2.3. Genetic Stability of HuN4-F112-eGFP

**Figure 4:**
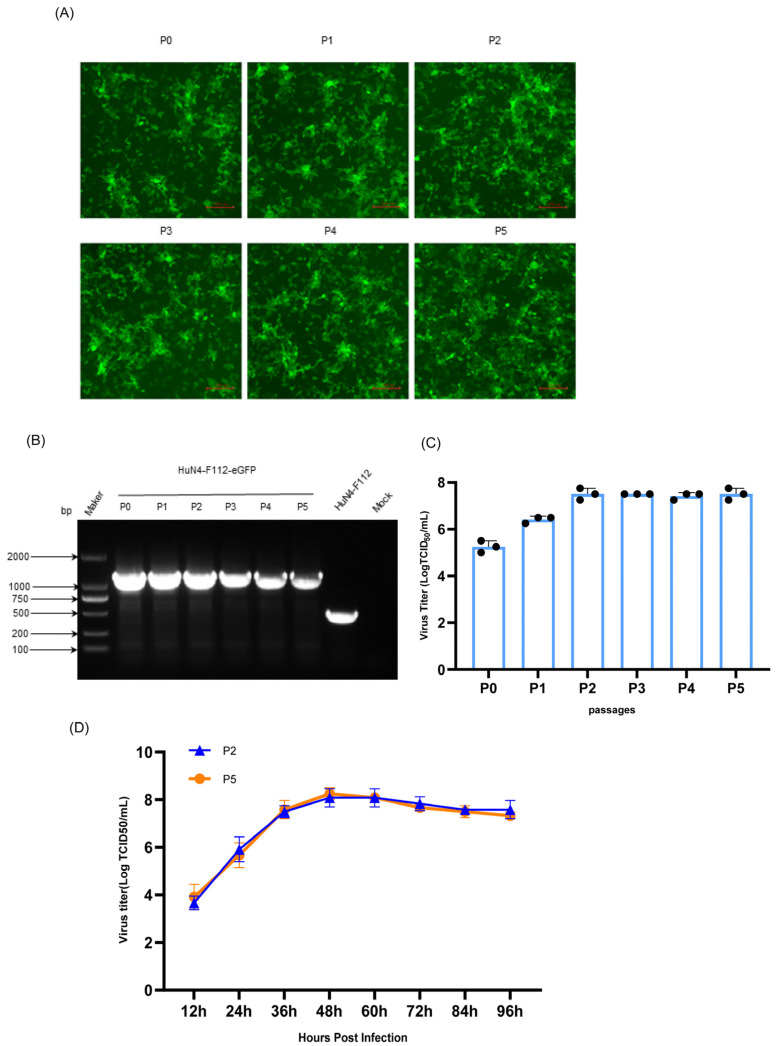
Genetic stability of the HuN4-F112-eGFP reporter virus. (**A**) The expression of eGFP in the MARC-145 cells infected with each virus passage. Scale bars represent 200 μm. (**B**) RNA extracted from cells infected with the P1–P5 viruses was analyzed using RT-PCR with F1 and R1 primers. The corresponding regions in the parental HuN4-F112 viruses were amplified as a control. The PCR products underwent visualization through 1% agarose gel electrophoresis. (**C**) The titers of the P0–P5 viruses were measured using the TCID_50_ assay. The number of black dots on the bars represents the number of replicates per data. (**D**) The growth kinetics of HuN4-F112-eGFP at the second and fifth passages were determined with viral titer tests. Results are means ± SD (triplicate).

To determine the genetic stability of HuN4-F112-eGFP, the rescued virus (P0) was serially passaged five times in MARC-145 cells using the end-dilution method to obtain the P0–P5 viruses. The green fluorescence expressions of the P0–P5 viruses remained mostly stable under a fluorescence microscope (Figure 4A). The sizes of the PCR products produced from the P0–P5 viruses were larger than those of the parental HuN4-F112 viruses (Figure 4B). As expected, sequencing results with the amplified fragments confirmed that no losses or mutations were detected in the eGFP gene. The viral titers tended to increase from P0 to P2 but became stable from P2 onwards (Figure 4C). The growth kinetics of the P2 and P5 viruses were analyzed, and both viruses showed similar growth trends, as shown in Figure 4D. Overall, HuN4-F112-eGFP remained stable during in vitro passages.

### 2.4. Development of a Sensitive HuN4-F112-eGFP-Based Neutralization Assay

Green fluorescence expressions could be observed in a single cell at an early time point (only one cycle of viral replication) after HuN4-F112-eGFP infection. The number of viruses could be determined by counting cells with green fluorescence, as each cell showing green fluorescent proteins was indicative of one virus at early infection. Therefore, an HuN4-F112-eGFP-based neutralization assay was designed to detect the presence of a virus in cells with greater sensitivity and accuracy, by identifying the virus after only a few cycles of viral replication.

**Figure 5:**
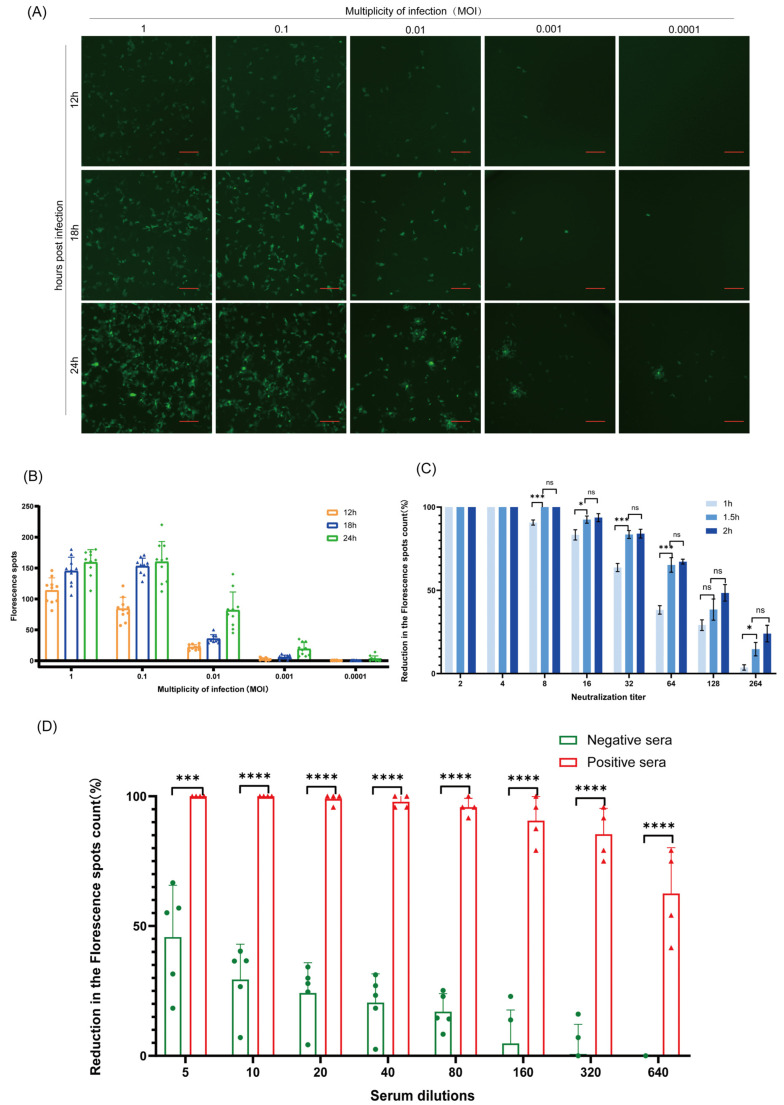
Development of a sensitive HuN4-F112-eGFP-based neutralization assay. (**A**) Fluorescent images of MARC-145 cells infected with HuN4-F112-eGFP over time. MARC-145 cells were cultured in 96-well plates and infected with HuN4-F112-eGFP at MOIs of 1, 0.1, 0.01, 0.001, and 0.0001. The cells were monitored for fluorescence expression under an inverted fluorescence microscope (Nikon, Japan) at 12, 18, and 24 h post-infection. Scale bars represent 200 μm. (**B**) GFP fluorescence dot count for images in (**A**). We counted the MARC-145 cells with GFP fluorescence in 10 wells for each viral infection dose at 12, 18, and 24 h post-infection using the counting method in Section 3.10. The number of dots on the bars represents the number of counting images. (**C**) Incubation time optimization. Each diluted serum was mixed with an equal volume of HuN4-F112-eGFP for periods of 1h, 1.5 h, and 2 h at 37 °C. The experiment was performed according to the procedure described in Section 3.7. The fluorescence inhibition ratio was compared for the different incubation times. ns *p* > 0.05, * *p* < 0.05, *** *p* < 0.001. (**D**) The effect of serum dilution on the neutralization assays. Four PRRSV-vaccinated pig sera and five negative pig sera were first pre-diluted, 1:5, in 0% DMEM, and then serially diluted two-fold with 2% DMEM. Then, virus neutralization assays were conducted, as previously described in Section 3.7. The fluorescence inhibition ratio was compared at different serum dilutions. *** *p* < 0.001, **** *p* < 0.0001. The number of dots on the bars represents the number of sera samples. All pig sera were heat-inactivated at 56 °C for 30 min. Error bars indicate the standard deviations.

To create an appropriate setting for the HuN4-F112-eGFP-based neutralization assay, the parameters, including virus MOI, serum dilutions, and incubation time, were optimized. At the 18th hour after infecting MARC-145 cells with HuN4-F112-eGFP at an MOI of 0.01, green fluorescence expression of individual cells, with a uniform cellular distribution, could be observed (Figure 5A). The data for the number of fluorescent dots exhibited a low level of discretization (Figure 5B). Therefore, the viral infection dose for the neutralization assay was set at an MOI of 0.01 and the result was observed at the 18th hour after infection. It was too late to observe the GFP expression at 24 hpi because the GFP expression spread to the surrounding cells (Figure 5A). For optimal neutralization, the incubation time serum dilution was incubated with the virus for 1.5 h at 37 °C (Figure 5C). There was a lack of adequate binding between the antigen and antibody during the 1 h incubation period. However, if the virus is left to incubate for 2 h, it may become less effective. When diluted in a 1:10 ratio with 0% DMEM, the negative porcine sera had a similar green fluorescence density compared with the virus-only sample, and showed less than 50% inhibition in the fluorescence expression of MARC-145 cells (Figure 5D).

Based on these initial studies, the serum samples underwent testing using the following general protocol. The sera were pre-diluted, 1:10, in 0% DMEM and heat-inactivated at 56 °C for 30 min to eliminate any remaining complement activity. The pretreated sera were serially diluted two-fold with 2% DMEM. Each serum dilution was incubated with an equal volume of HuN4-F112-eGFP (0.1 MOI) for 1.5 h at 37 °C. The remaining experimental operations were performed as described previously.

### 2.5. Comparison of the Neutralization Titers Assayed with the CPE and eGFP Methods

**Figure 6:**
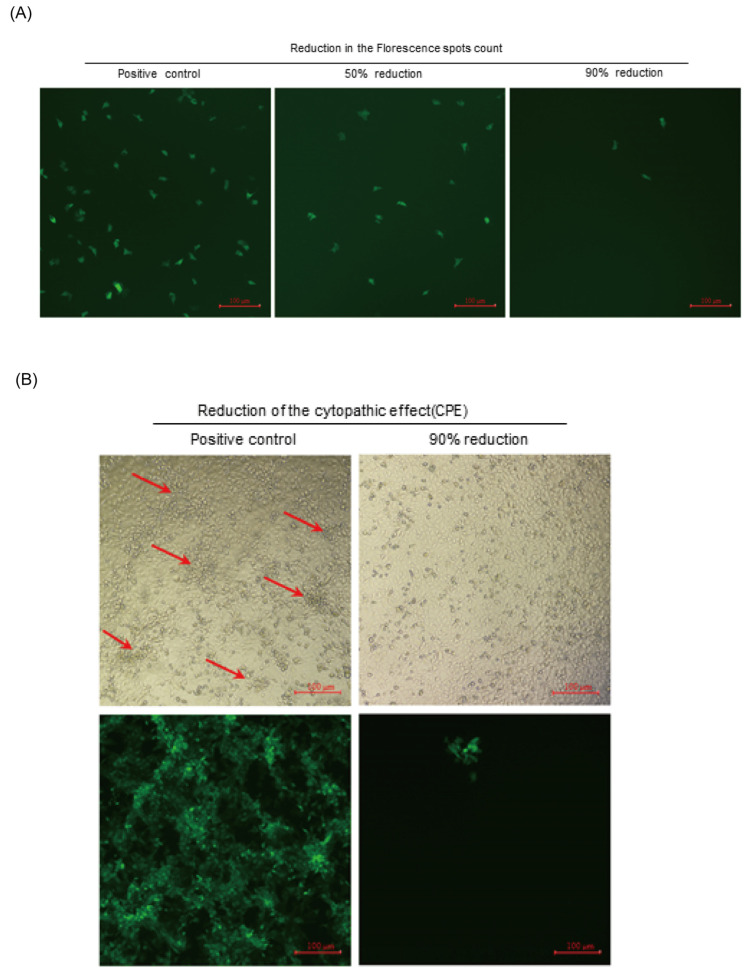
A comparison of the HuN4-F112-eGFP-based neutralization assay with the traditional CPE reduction assay. (**A**) Fluorescent images were captured to record the number of cells with green fluorescence in the field of view at 18 hpi using the HuN4-F112-eGFP-based neutralization assay. Scale bars represent 100 μm. (**B**) Images were captured to record the cytopathic effect (CPE) at 72 hpi using the CPE-based neutralization assay under bright and GFP fields. The red arrows sign the appearance of CPEs. Scale bars represent 100 μm.

In total, 27 pig sera, collected from three pigs involved in vaccine studies, were investigated for neutralization titers against HuN4-F112 using a sensitive HuN4-F112-eGFP-based neutralization assay, as well as the traditional CPE reduction assays. Images of green fluorescence were captured to record the number of cells with green fluorescence in the field of view at 18 hpi (Figure 6A). The CPE-based neutralization assay was used to examine neutralization levels at 72 hpi (Figure 6B). Different neutralization abilities were observed among serum samples, as shown in Table 1 for virus neutralization titers. Both methods yielded titers with similar trends, and the eGFP-based assay resulted in higher antibody titers than the CPE-based method on each sample, which showed that the newly developed assay has the advantage of a one- to four-fold increase in sensitivity at the early stage of antibody response. The CPE-based method relies on a CPE to establish the level of observability. This process can be time-consuming, as it requires waiting for the results. The assay in this study detected that viral replication could be determined by eGFP expression, which shortened the endpoint observation time of the neutralization assay to as early as 18 hpi, thus saving 2 days of assay waiting time.

### 2.6. HP-PRRSV Neutralizing Antibody Response Patterns Detected via the GFP Neutralization Assay

**Figure 7:**
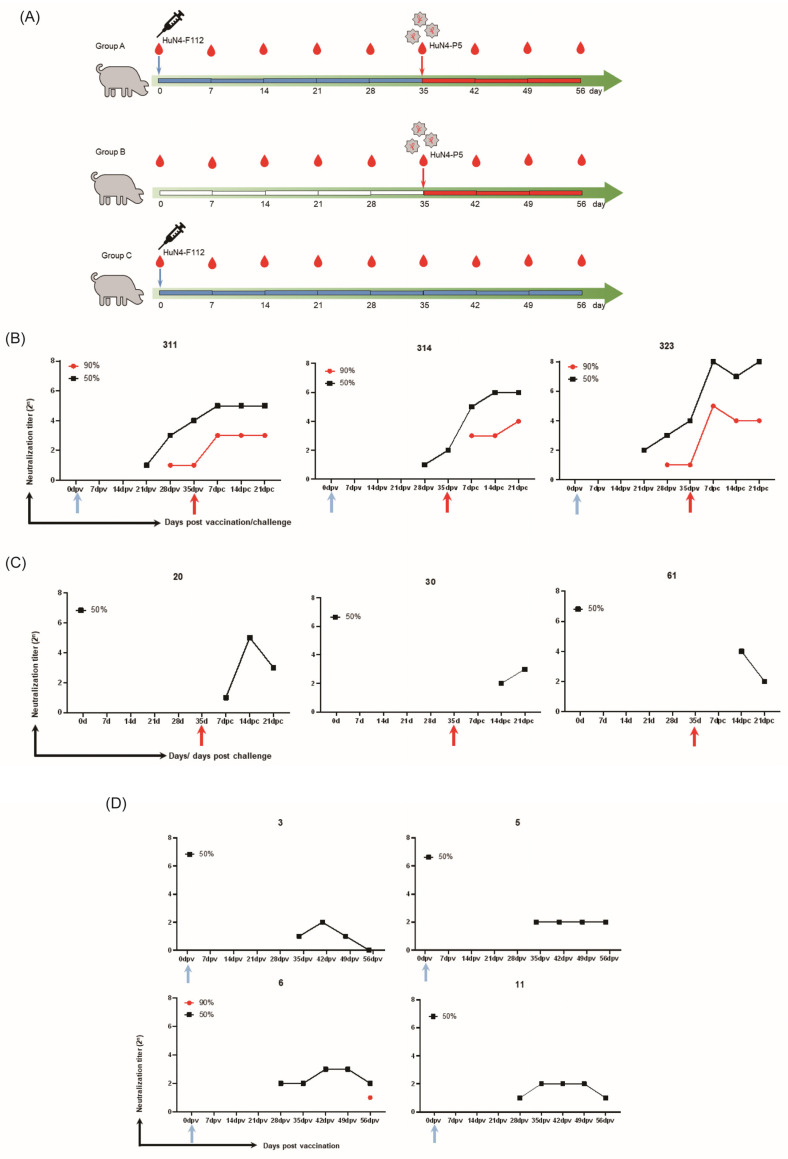
HP-PRRSV neutralizing antibody response patterns detected using a GFP neutralization assay. (**A**) All pig sera were divided into three groups. Group A included sera from three pigs that were injected with the HuN4-F112 virus on day 0 and challenged with HuN4-P5 at 35 days post-vaccination. Group B included sera from three pigs that were only challenged with HuN4-P5 on the 35th day. Group C consisted of sera from four pigs that were injected with the HuN4-F112 virus on day 0. (**B**–**D**) The neutralization titers of serum samples from each piglet in Group A (**B**), Group B (**C**), and Group C (**D**), collected at 0, 7, 14, 21, 28, 35, 42, 49, and 56 days post-vaccination. Immunized piglets showed faster and stronger NAb responses after a virulent challenge. Blue arrows suggest that pigs were injected with the HuN4-F112 virus on 0 dpv, and red arrows suggest that pigs were challenged with HuN4-P5 on 35 dpv.

All serum samples from animal experiments for HP-PRRSV vaccine studies were divided into three groups, as described in Section 3.2 (Figure 7A). All piglets in Groups A and C were PRRSV antibody-positive 14 days post-vaccination and did not exhibit any clinical signs following immunization with HuN4-F112. Following the challenge, Group A piglets exhibited no clinical signs, whereas Group B piglets displayed persistent high fevers and several clinical symptoms, including loss of appetite, loss of liveliness, and breathing complications. Furthermore, the levels of PRRSV antibodies in the piglets of Group B rapidly increased after the challenge. The clinical manifestation showed that vaccinated animals exhibited protection against the challenge.

NAbs were detected in all pigs in Group A (pigs numbered 311, 314, and 323) at 4 weeks post-vaccination (Figure 7B). Moreover, as early as 3 weeks post-vaccination, low levels of NAbs could be detected in the pigs numbered 311 and 323. The levels of NAbs rose rapidly over time following the challenge with HP-PRRSV HuN4-F5 (Figure 7B). In Groups B and C, only the neutralization titers that induced 50% inhibition in piglet serum could be detected after vaccination or challenge, and the levels were very low (Figure 7C,D). Immunized piglets demonstrated faster and stronger NAb responses after a virulent challenge (Figure 7). The levels of PRRSV NAbs and anti-N protein antibodies were inconsistent, and NAbs were produced much later than normal antibodies.

## 3. Materials and Methods

### 3.1. Cells and Viruses

African green monkey kidney cells (MARC-145 cells), kept in our laboratory, were grown in Dulbecco’s modified Eagle medium (DMEM) (Thermo Fisher Scientific, Waltham, MA, USA), supplemented with 10% fetal bovine serum (FBS) (Thermo Fisher Scientific, Waltham, MA, USA) at 37 °C in a 5% CO_2_ atmosphere. HuN4-F112, a strain of Type 2 PRRSV, is a live-attenuated vaccine that was derived through the consecutive passage in MARC-145 cells from HP-PRRSV HuN4 (GenBank accession no. EF635006). A full-length cDNA clone of HuN4-F112, preserved in our laboratory, was used as the backbone for inserting the eGFP gene (GenBank accession no. EU056363.1) [31].

### 3.2. Pig Sera

All pig serum samples used in our research were obtained from animal experiments for HP-PRRSV vaccine studies. All experimental protocols followed the guidelines for the care and use of animals. They were approved by the Ethical Committee of the Shanghai Veterinary Research Institute, Chinese Academy of Agricultural Sciences, on 22 May 2023, under the number SV-20230519-01. Ten 30-day-old piglets were purchased from a breeding farm in Shanghai, China. The animals were confirmed to be negative for PRRSV using RT-PCR and confirmed PRRSV antibody-negative using an ELISA (IDEXX, USA). Three piglets were injected intramuscularly on day 0 with 2 mL of diluted HuN4-F112 virus (1 × 10^5^ TCID_50_), and then challenged with HuN4-P5 on the 35th day (Group A). Three pigs were infected with HuN4-P5 on the 35th day, to serve as a control group (Group B). In addition, four piglets were only vaccinated on day 0 with the HuN4-F112 virus (1 × 10^5^ TCID_50_) (Group C). Serum samples from each piglet were collected at 0, 7, 14, 21, 28, 35, 42, 49, and 56 days post-vaccination to detect NAbs.

### 3.3. Construction of a Recombinant PRRSV Full-Length cDNA Clone Harboring eGFP

To construct an eGFP-tagged recombinant virus, a strategy (Figure 1) was designed to insert the coding sequence of the eGFP protein into the PRRSV genome. The inserted fragment included the eGFP gene and two restriction sites (Asc I and EcoR V), and it was commercially synthesized through DNA synthesis technologies (Tsingke Biotech Co., Ltd., Beijing, China). Using the commercially synthesized fragment as a template, F1 (5′-AGTGCATACCATGGTGAAATGCCTC-3′) and R1 (5′-TGAACGGTAGAGCGCGCACGGAGTA-3′) primers were used to amplify the inserted fragment. The full-length cDNA clone HuN4-F112 and the amplified products were digested with Asc I and EcoR V and ligated with T4 DNA ligase. The eGFP gene was inserted between the ORF1b and ORF2a genes of the HuN4-F112 infectious clone. In addition, the recombined plasmid HuN4-F112-eGFP was confirmed through restriction mapping and Sanger sequencing.

### 3.4. Rescue of the Recombinant Virus

The recombined plasmid HuN4-F112-eGFP was linearized with restriction enzyme SwaI, which cuts downstream of the poly (A) tail. According to the manufacturer’s instructions, linearized templates were purified and used for in vitro transcription using the mMESSAGE mMACHINE™ SP6 Transcription Kit (Thermo Fisher Scientific, Waltham, MA, USA). The synthetic RNA was transfected into MARC-145 cells in a six-well plate at 80% confluence using DMRIE-C reagent (Thermo Fisher Scientific, Waltham, MA, USA), according to the manufacturer’s description. The cells were maintained in Eagle’s minimum essential medium (EMEM), containing 2% FBS, 6 h post-transfection. At the same time, the normal MARC-145 cells were operated in parallel, serving as a negative control. The cells were monitored for fluorescence expression under an inverted fluorescence microscope (Nikon, Tokyo, Japan) every day. Cell culture supernatants were harvested when 100% of the cells demonstrated fluorescence expression and were serially passaged on MARC-145 cells. Viral RNA was extracted from the pellet using an RNeasy kit (QIAGEN, Duesseldorf, Germany). F1 and R1 primers and first-strand cDNA synthesized with the SuperScript III First-Strand Synthesis System (Thermo Fisher Scientific, Waltham, MA, USA) were used to amplify the replaced or inserted regions and their flanking areas of viruses. The purified PCR products were used for sequencing. Meanwhile, the infected cell lysates were collected at 20 h post-infection (hpi) to detect N protein expression via Western blotting.

Furthermore, to determine the biological characteristics of HuN4-F112-eGFP in vitro, the growth curves of HuN4-F112-eGFP (the 5th passage) and the parental virus HuN4-F112 were determined. MARC-145 cells were infected with HuN4-F112-eGFP/HuN4-F112 at a multiplicity of infection (MOI) of 0.01. After 1 h of virus absorption, the cell culture supernatant with the HuN4-F112-eGFP virus was replaced with DMEM with 2% FBS. The multiple-step growth curves within 96 h post-infection were determined with viral titers and tested using the Reed–Muench method.

The plaque morphology was also determined in MARC-145 cells, as described below. The MARC-145 cells were incubated in 6-well plates with 10-fold diluted HuN4-F112-eGFP or HuN4-F112 for 2 h, then washed twice with 1 × PBS, and then overlaid with 3 mL of MEM containing 2% FBS (Sigma-Aldrich, St. Louis, MO, USA) and 1% UltraPure™ low-melting-point agarose (ThermoFisher Scientific, Waltham, MA, USA). At 3 dpi, the cells were fixed with 4% paraformaldehyde and stained with 0.1% crystal violet to detect plaques.

### 3.5. Genetic Stability of HuN4-F112-eGFP during Serial Passages in MARC-145 Cells

The HuN4-F112-eGFP viruses (P0) were serially passaged 5 times in MARC-145 cells, using the end-dilution method to obtain the P0–P5 viruses. For the stability of eGFP expression in different virus passages (P0–P5), green fluorescence was observed at 24 hpi under a fluorescence microscope. The eGFP genes in the P0–P5 viruses were identified via gel electrophoresis of PCR products and sequencing analysis, as described above. The Reed–Muench method was used to test the viral titers of the P0–P5 viruses. Meanwhile, the growth kinetics of HuN4-F112-eGFP at the 2nd and 5th passages were determined using viral titer tests.

### 3.6. Exploration of Viral Infection Dose for a Neutralization Assay

MARC-145 cells in 96-well plates were infected with HuN4-F112-eGFP at different viral infection doses (1, 0.1, 0.01, 0.001, and 0.0001 MOI). The cells were monitored for fluorescence expression under an inverted fluorescence microscope (Nikon, Tokyo, Japan) at 12, 18, and 24 h post-infection. Meanwhile, the MARC-145 cells with GFP fluorescence were counted for each viral infection dose, using the counting method in Section 3.10. Their green fluorescence expressions could be observed in a single cell, and these cells had a uniform distribution under the inverted fluorescence microscope.

### 3.7. PRRSV-eGFP Neutralization Assay

Pig serum samples were obtained from HuN4-F112 vaccine studies in our previous study. The pretreatment sera were serially diluted 2-fold with 2% DMEM. Each serum dilution was incubated with an equal volume of HuN4-F112-eGFP (the 5th passage, 1000 TCID_50_/well, 0.01 MOI) for 1.5 h at 37 °C. The mixtures (100 μL/sample) were placed on the confluent MARC-145 cell monolayers in a 96-well plate. The medium was added without serum in control wells. After incubation at 37 °C for 18 h in 5% CO_2_, the numbers of cells with GFP fluorescence were counted under an inverted fluorescence microscope (Nikon, Tokyo, Japan). The neutralization titers were taken as the reciprocal of the dilution that induced 50% or 90% inhibition of fluorescence expression of MARC-145 cells in a 96-well plate.

### 3.8. Pretreatment of Pig Serum Samples and the Optimization of Incubation Time

The pig sera were heat-inactivated at 56 °C for 30 min to remove residual complement activity. Four PRRSV-vaccinated pig sera and five negative pig sera were first pre-diluted, 1:5, in 0% DMEM, and then serially diluted 2-fold with 2% DMEM. Then, virus neutralization assays were conducted, as previously described in Section 3.7. The inhibition ratio of the fluorescence expression of MARC-145 cells in a 96-well plate was calculated at different serum dilutions.

To optimize the incubation time, each diluted serum sample was mixed with an equal volume of HuN4-F112-eGFP for 1 h, 1.5 h, and 2 h at 37 °C. The experiment was performed according to the procedure described previously in Section 3.7. The inhibition ratio was calculated for the different incubation times.

### 3.9. Cytopathic Effect (CPE) Reduction Assay

A CPE reduction assay was performed based on the PRRSV-eGFP neutralization assay previously described. However, the HuN4-F112-eGFP infection dose was 200 TCID_50_/well in a 96-well plate. The mixtures (100 μL/sample) were placed on the confluent MARC-145 cell monolayers in a 96-well plate. The MARC-145 cells were examined for a CPE at 72 h post-inoculation. The neutralization titers were expressed as the reciprocals of the dilution that induced 90% inhibition of the appearance of a CPE.

### 3.10. Data Analysis

Fluorescence images were acquired from three random regions at the same exposure intensity and position in each well. Image J software (V1.8.0.112) was employed to count the MARC-145 cells with GFP fluorescence for each picture. The inhibition ratio = (1 − (mean plaques in sample)/(mean plaques in control)) × 100%.

## 4. Discussion

In this study, we generated a recombinant PRRSV full-length cDNA clone harboring the eGFP gene, which was inserted between the ORF1b and ORF2a genes. The recombinant PPRSV HuN4-F112-eGFP was successfully recovered in MARC-145 cells with reverse genetics. The eGFP gene insertion had some effect on the growth kinetics of HuN4-F112-eGFP, for which it exhibited lower titers than for parental HuN4-F112. The fluorescent protein expressions of the reporter virus HuN4-F112-eGFP remained stable for at least five passages.

Following the first successful use of reverse genetics for PRRSV-1 in 1998 [32], reverse genetics gradually became a valuable tool in PRRSV studies. PRRSV has been investigated as a viral vector for expressing a desired foreign gene in recent years. Several PRRSV reporter viruses, expressing luciferase and clover, have been generated for antiviral drug screening and serum neutralization assays [23,25,28]. There are two primary methods used for introducing a foreign gene into PRRSV. One is to incorporate the gene as a fusion protein within nsp2 [8,33,34]. However, reporter genes inserted into the PRRSV genome using this strategy exhibit genetic instability [25]. The other method involves inserting an additional subgenomic RNA into the PRRSV genome at one of the three following potential locations: between ORF1b and ORF2a [26,28,29,35], between ORF4 and ORF5a [36], or between ORF7 and 3′UTR [37]. The cytokine GM-CSF and the E2 protein of CSFV were inserted between the ORF1b and ORF2a genes in HuN4-F112 genomes. Both were stably expressed in cells infected with the recombinant virus after at least 20 passages [26,29]. Therefore, the eGFP gene was inserted between ORF1b and ORF2a genes in HuN4-F112 genomes to guarantee genetic stability, as stability is a prerequisite for a good viral reporter system.

The neutralization assay, based on HuN4-F112-eGFP, was able to detect the presence of a virus in cells with greater sensitivity and accuracy. Each cell showing green fluorescent protein was indicative of one virus at the early infection stage. Therefore, the number of viruses in the early infection stage could be determined by counting the cells showing green fluorescent protein. In order to ensure a more standardized and robust assay, the residual complement was removed as soon as possible [38]. We found that negative porcine serum in low dilutions could partially inhibit viruses during the experiment (Figure 5D); thus, all pig sera were heat-inactivated at 56 °C for 30 min and diluted in a 1:10 ratio to remove residual complement activity. ImageJ software (V1.8.0.112) was used to batch count the MARC-145 cells exhibiting GFP fluorescence in each image and render the assay less labor-intensive so that it would be more suitable for standardization and high-throughput purposes. The current assay detected that the viral replication could be determined through eGFP expression, which shortened the endpoint observation time of the neutralization assay to as early as 18 hpi, without requiring fixation, staining, or manual counting of plaques. Meanwhile, the assay has a reduced reliance on advanced instruments such as the fluorescence Elispot reader [38] and the ArrayScan system [39]. Further exploration is needed to determine if this assay based on HuN4-F112-eGFP is effective for other PRRSV species, as this study was specific to the PRRSV Type 2 virus strain.

Anti-N protein antibodies can be detected with the IDEXX PRRSV test 7–14 days after early PRRSV vaccine (HuN4-F112) immunization. However, the presence of specific antibodies and traces of PRRSV in the serum suggests that the early antibody response does not provide protection against PRRSV infection [15,40,41,42]. There is experimental evidence suggesting that the onset of NAbs is accompanied by clearance of the virus from circulation and tissues after infection [43,44]. It has been reported that PRRSV-inactivated vaccines against PRRSV that did not induce NAbs were ineffective, while attenuated vaccines that induced some levels of NAbs were effective [15]. Therefore, NAbs play a crucial role in safeguarding against PRRSV infection. However, NAbs are specific to the vaccine strain, and there are lower or no titers for cross-NAbs (heterologous) [45,46]. In this study, priming of immunity, through either a single virulent challenge or only vaccination, was able to produce limited NAbs, with titers remaining low until the end of the study period (Figure 7C,D). Piglets immunized with HuN4-F112 could induce a faster and stronger NAb response after a virulent challenge from HuN4-F5 (Figure 7). Multiple vaccinations or infections are able to produce sufficient levels of NAbs for protection, similar to the results of certain other works [45,47]. In addition, PRRSV NAbs appear slowly and inconsistently, at periods equal to or later than 4 weeks post-exposure to PRRSV [44]. The risk of reversion to virulence raises some concerns about whether its utilization through attenuated vaccines is effective. Scientists and veterinarians are currently exploring new strategies for vaccine design. An ideal vaccine against PRRSV should rapidly induce a high level of NAbs, as well as a specific cellular response against the virus.

## 5. Conclusions

In summary, we successfully rescued a stable PRRSV expressing the eGFP gene based on the infectious clone HuN4-F112-eGFP, which we constructed. Based on this reporter virus, a neutralization assay could easily be used to evaluate the level of NAbs by counting cells with green fluorescence, which shortened the endpoint observation time of the neutralization assay to as early as 18 hpi. The recombinant PRRSV may represent a powerful tool for developing vaccines or screening antivirals and investigating antibodies serologically. The novel HuN4-F112-eGFP-based neutralization assay holds the potential to provide a highly efficient platform for evaluating the next generation of PRRS vaccines.

## Figures and Tables

**Table 1:** Neutralization titers of pig sera tested with the HuN4-F112-eGFP-based neutralization assay and the traditional CPE reduction assay.

		Reduction in Fluorescence Spots Counts ^a^						90% Reduction in the Cytopathic Effect (CPE) ^b^		
Sample Number		31		32		33		31	32	33
	Percentage	90%	50%	90%	50%	90%	50%			
Days post vaccination/challenge										
0 dpv		/	/	/	/	/	/	/	/	/
7 dpv		/	/	/	/	/	/	/	/	/
14 dpv		/	/	/	/	/	/	/	/	/
21 dpv		/	2	/	/	/	4	/	/	/
28 dpv		2	8	/	2	2	8	/	/	/
35 dpv		2	16	2	4	4	16	2	/	2
7 dpc		8	32	8	32	32	256	4	4	8
14 dpc		8	32	8	64	16	128	4	4	8
21 dpc		8	32	16	64	32	256	8	8	16

The neutralization titers were taken as the reciprocals of the dilutions. ^a^ Fluorescence (eGFP) reading at 18 h post-inoculation. ^b^ CPE reading at 72 h post-inoculation.

## Data Availability

Data are contained within the article.
